# Chicago Medical Society, November 16th

**Published:** 1885-12

**Authors:** 


CHICAGO MEDICAL SOCIETY.

*Stated Meeting, November 16th, 1885.*—The PRESIDENT, C. T. PARKES, M. D., in the chair.

A REPORT EMBODYING TWO HUNDRED CASES OF TONSILITIS, was the title of a paper read by DR. J. M. G. CARTER, of Waukegan, Illinois.

After detailing the treatments which had been employed in the cases, the author advanced the theory that since the great majority of the cases occurred during March, April and May, when northeast winds prevailed, carrying landward more moisture from Lake Michigan, these winds must be one of the casual factors in the production of tonsilitis. Also, the test for ozone during these months showed a greater percentage of ozone in the atmosphere. Another fact was noticed, that a great many cases occurred in rheumatic patients. The author was of the opinion that the epidemic of cases detailed was due to the damp northeast winds, containing an excess of ozone, and to unusually disturbed electrical conditions of the atmosphere. The author grouped the two hundred cases, without classifying them, into cases of simple, diphtheritic or scarlatinous tonsilitis. He mentioned, however, the fact that cases of simple tonsilitis were often accompanied or followed by attacks of diphtheria or scarlet-fever among other members of the family.

DISCUSSION.

PROFESSOR F. O. STOCKTON said: We have tonsilitis in nearly all the eruptive fevers, usually following, and occasionally preceding them. The author refers to cases having diphtheritic patches on the tonsils; I think he has confounded these diphtheritic cases with what we specialists call follicular tonsilitis. It is not a diphtheritic condition at all, but resembles it very greatly, so that in a differential diagnosis these cases are very often confounded. In regard to the temperature going up to 104, it is my experience, and that of authorities such as Cohen, Bosworth and Robinson, that it ranges from 102 to 103, seldom more than 103, usually 102. In the treatment of tonsilitis I have never found a gargle effective In diphtheria or acute tonsilitis you can give chlorate of potash, or any other drug, but where a man has a pain in the angle of his jaw and a throat so sore he cannot swallow, if you can get him to gargle you can do more than I can. Ice held in the mouth until it dissolves is good, also powder or tincture of guaiac and tincture of aconite internally, but never give more than three doses of aconite in one day. Occasionally, if you see a case is going on to suppuration, use hot applications externally and internally in the way of steam, otherwise never use hot applications. My idea is that heat promotes congestion more than cold, and my experience is that where cases are treated, some with cold and others with hot applications, that the cold in connection with other treatment, guaiac and tincture of aconite, is most successful. We ought to settle this question of tonsilitis. What is acute tonsilitis, and is there such a thing as acute tonsilitis? To me it is a misnomer. The abscess seldom forms in the tonsil, it is beind it in the loose connective tissue; and in opening it we cut behind or above the tonsil.

PROFESSOR C. W. EARLE said: I would like to ask Dr. Carter if there was any appearance of contagion in any of the cases?

DR. J. M. G. CARTER said: Frequently the disease will attack everybody in the family, old and young, and just as frequently but one or two in the family will have the disease. It sometimes appears to be contagious.

PROFESSOR G. C. PAOLI said: Tonsilitis is a very common disease in Chicago, especially among children. It is certain that there are different degrees of tonsilitis; there are light cases in which very little medicine is required, and again there are strumous children who are very susceptible to the changes of weather, and in inclement weather get wet and have tonsilitis. In some cases one tonsil is affected, in others both, or the pharynx. There are grave cases that cannot be cured in one week, or two, cases that develop into hypertrophy of the tonsils. In regard to treatment: a light case does not require ice, and a person with malignant scarlatina with tonsils affected would take cold from the application of ice. We should discriminate, and in malignant cases where the tonsils are inflamed should be careful about applying ice. In the use of aconite with children we should be very careful, as in fever it diminishes the circulation, and I would not recommend its general use unless you can see the patient two or three times a day and watch the effect of the aconite. A simple thing to use is a little potash with tincture of iodine. In scarlatina complicated with diphtheria it is better to use very little medicine. I have nothing against chlorate of potash, however I read a very interesting paper by a professor in New York, his name I do not now remember, who says that observation has shown that chlorate of potash has often produced nephritis, and I think great care should be taken in its use.

DR. R. TILLEY said: One of the points brought forward by the author is the use of kerosene. I remember that Besnier, a professor of skin-diseases in Paris, says that kerosene is used extensively by the laity, but he regards it as a dangerous remedy in the hands of people generally, and a very inefficient one; he was speaking, it is true, of the treatment of itch. I protest against the use of the terms ozone and electrical conditions of the atmosphere: we know practically nothing about either.

DR. J. M. G. CARTER said that he was obliged to leave in order to take the train for Waukegan, and expressed regret that he could not remain and reply to the criticisms upon his paper.

PROFESSOR W. E. QUINE said: An interesting feature of the experience embodied in the paper is the obvious failure of the writer to differentiate infectious from non-infectious tonsilitis. I presume it is a matter of familiar observation to all who have been long engaged in the practice of medicine that many cases of follicular tonsilitis occur which baffle the judgment of the most experienced physician to determine with precision whether they are infectious or non-infectious. I have often seen in my own practice cases of this kind. Often one member of a family, probably the first one attacked, exhibits plainly marked features of simple follicular tonsilitis, and those of the family who sicken afterwards exhibit the phenomena of diphtheria, or less frequently, scarlatina. The text-books do not give a reliable guide to diagnosis, and if any of my colleagues know of means by which cases of this kind can be differentiated with certainty we would like to know them.

One gentlemen has alluded to the opinion of an eminent professor in New York; I remember that Jacobi, who is perhaps the person referred to, in a recent article maintains very vigorously that many cases of so-called tonsilitis are in reality immature cases of diphtheria, and he stoutly maintains that there are many cases of diphtheria never having patches in the throat, and where the patient walks on the street and communicates the disease freely to those with whom he comes in contact.

PROFESSOR SARAH HACKETT STEVENSON said: Last February I was called to see a lady who had frequently suffered from tonsilitis. She was "subject to quinsy." I suggested that her child should not be kept in the same room with her as the case seemed more than ordinarily violent, and gave the usual treatment for quinsy; the disease went on to suppuration. I was called out of the city after one of the tonsils had discharged, and during my absence, about five days, her child was attacked with malignant scarlet fever, and died before my return. This is the first case in which I ever suspected that a benign form of tonsilitis might reproduce a malignant form. Since then I have watched all cases, however simple they may seem.

DR. C. T. FENN said: I think we should all take an interest in this work, especially in the most practical suggestions of Dr. Earle. The fact is, that to regard these cases all as malignant, is to be on the safe side. A case of simple tonsilitis has a tendency to develop into diphtheria. I protest against the folly of attempting to distinguish between mild and simple cases of tonsilitis and diphtheria. In regard to treatment, I have no use for gargles or washes; I will not force open the mouth of a child and cause it to cry, but steadily and persistently, every fifteen minutes, I will give such medicines as the child will pass over the tonsils when swallowing.

PROFESSOR J. S. KNOX said: I regret that the paper is so general, the author evidently groups together a variety of cases of tonsilitis of different classes. There is undoubtedly an inflammation of the tonsils due to eruptive diseases, such as small-pox and scarlet fever, and there is a tonsilitis which is purely catarrhal, and another which is purely due to diathesis and which is rheumatic or syphilitic. The treatment differs accordingly. I think there is an error as to the value of chlorate of potash; better results will be obtained from the use of bicarbonate of potash, the value of the drug lying in the fact that it is a potash salt, and I think that the bicarbonate of soda would be still much more efficient. Where the hyposulphite of soda would do good, the salicylate would do more.

DR. J. J. M. ANGEAR said: It seems to me that if we remember that there is such a thing as resistance, the absence of which is susceptibility, it will explain some, if not all of these difficulties. We can readily imagine a robust, healthy child with strong resistance to morbid influences, and especially that of diphtheria, on exposure, would have simple tonsilitis (abortive diphtheria); but suppose that his brother, with strong susceptibility, is exposed to the same morbid influences, he will develop a case of undoubted diphtheria.

In diphtheria, we have an inflammation with fibrinous exudation which breaks down the mucous cells and forms the diphtheritic patch; this furnishes a nidus for the micro-organisms, which go on secreting or fermenting their peculiar virus, the absorption of which contaminates the whole body, and now we have a constitutional disease. Will not this explain a large number, if not all, of those cases where four or five children in a family are taken down apparently with simple tonsilitis, and some one child that has not their resistance is attacked with undoubted diphtheria. In this house we have tonsilitis, and our neighbor severe diphtheria. By remembering these pathological facts, we shall see that it is, or it may be, all the same morbid influence here and yonder,—here, recovery in a few days; there, death in a few hours.

PROFESSOR F. O. STOCKTON said: In acute tonsilitis I think it will be found that ice is the proper treatment in the first stage, before suppuration has begun. It is very seldom that pus is located in the tonsil, it is behind the tonsil. With regard to a differential diagnosis between diphtheria and tonsilitis, there is almost always in diphtheria a regularly graded rise in the temperature; in acute tonsilitis, so-called, or follicular, the temperature is not regular, it rises at a jump, the attack comes on suddenly, begins with a chill immediately followed by fever; in diphtheria there is a gradual rise, going up one day, dropping the next. I think if we took a record of given cases of diphtheria and tonsilitis, we would find that a regular rise in temperature in diphtheria occurs as in the essential fevers.

